# Prevalence of Type I Lip Print among Medical Students in A Medical College of Nepal

**DOI:** 10.31729/jnma.4451

**Published:** 2019-08-31

**Authors:** Sharmila Gurung, Vijay Gupta, Anita Lamichhane

**Affiliations:** 1 Department of Forensic Medicine and Toxicology, Devdaha Medical College and Research Institute, Rupandehi, Nepal; 2 Department of Forensic Medicine and Toxicology, Nepalese Army Institute of Health Sciences, Kathmandu, Nepal; 3 Department of Pediatrics, Lumbini Medical College, Pravas, Palpa, Nepal

**Keywords:** *cheiloscopy*, *forensic odontology*, *lip prints*, *personal identification*

## Abstract

**Introduction:**

Lip prints, due to their unique patterns are typical to an individual, hence, used for personal identification. They vary in predominance, gender and race among different populations. The objective is to study their distribution among medical students and identify the predominant type.

**Methods:**

The descriptive cross-sectional study was conducted on the medical students of Nepalese Army Institute of Health Sciences, from April 2019 to May 2019 after the ethical approval. Convenience sampling was used. The prints were classified according to Suzuki and Tsuchihashi. The collected data was entered in SPSS to determine the frequency and percentage. Sub group analysis was done on basis of gender and types of lip print.

**Results:**

Out of 205 participants, prevalence of type I lip print among medical students is 70 (34.1%). Type I lip print was found to be most common followed by Type II in 57 (27.8%) and Type V in 6 (2.9%). One hundred forty one (68.8%) were male and 64 (31.2%) were female. The predominating pattern in RUQ; LUQ; LLQ; RLQ among male and female is Type I 46 (32.6%) and Type I 39 (27.7%); Type II 39 (27.7%) and Type II 44 (31.2%); Type I 19 (29.7%) and Type II 19 (29.7%); Type I 31 (48.4%) and Type I 27 (42.2%) respectively.

**Conclusions:**

Type I was the most common while Type V was the least common lip print. However, there was variation in its frequency and distribution according to the quadrant and sex.

## INTRODUCTION

Lip prints are patterns formed by lines or fissures along the outer surface of the lips and their study is known as cheiloscopy. It is a well-accepted tool for human identification, an essence of any forensic investigations.^1^ Lip prints are individualistic and persistent. Hence, it is a reliable tool for personal identification. Establishing identity poses challenge because visual identification of a person can be difficult, unreliable or even impossible at times, while fingerprints may be intentionally avoided.

However, lip prints left on various articles in latent or conventional form, can provide information sufficient for positive identification.^2^ They become indispensable but vary in predominance, gender and race.^3–5^ More studies on lip prints in relation to sex for its utility as sex determination are recommended.^6^

The study was conducted to assess the distribution of types of lip print among medical students and identify the predominant type in either sex.

## METHODS

This descriptive cross-sectional study was conducted in Nepalese Army Institute of Health Sciences (NAIHS) from April 2019 to May 2019. Ethical approval was received from the institutional review committee (IRCNAIHS) and the concerned authority to conduct the study. The study population is the medical students of the same medical college. The students without any anatomical deformity, lesion on the transition zone of the lips on the lips were included and those with known hypersensitivity to lipsticks were excluded from the study. The study protocol and objectives were thoroughly ex plained to the participants and written informed consent was obtained.

The participants were asked to thoroughly clean their lips, followed by application of non-glossy, nonpersistent red colored (golden rose velvet matte 11) lipstick over the whole lips. They were then asked to gently rub their lips together for even distribution of the lipstick. A white A4 size print paper was folded in half and pressed between the lips to record the lip prints. During the procedure, they were refrained from further moving their lips so as to avoid any distortions on the print. The print was repeated in case of any defect. Once, confirmed, their participants cleared their lips with wet wipes and cleansing milk. The record was then preserved by applying a strip of cellophane tape from its center, sideways spread. It was then assigned a serial number and labelled with details such as name, age and sex. The print was divided into four quadrants by a horizontal line from one angle of the mouth to the other and a center line, from the tip of the philtrum, thus forming the right upper quadrant (RUQ), left upper quadrant (LUQ), right lower quadrant (RLQ) and left lower quadrant (RLQ). The lip prints were then examined by a magnifying glass in each quadrants, for their shape and pattern and classified according to Tsuchihashi's classification,^7^ which is the most commonly used classification worldw ide. Every record was independently studied by the researchers to assure the validity and reliability of the classification proc ess.

Sample size for finite population was calculated using the formula,


$$\begin{array}{l} \text{X=}\underline{{\text{Z}}^{2}}\underset{{\text{d}}^{2}}{\underline{\text{xp}\left(\text{1-p}\right)}} \end{array}$$

where,

Z=1.96 at 95% CI
p= prevalence of lip print, 75% [Educated guess]
q= 1-p
d= margin of error, 6%

Total sample size was calculated to be 200. The data was then entered and assessed for frequency and percentage for the variables through IBM SPSS statistics 20 software.

## RESULTS

Among 205 participants, prevalence of Type I lip print is 70 (34.1%), which was the commonly occurring pattern in our study and was present in almost all quadrants except the left upper quadrant, which was followed by Type II 57 (27.8%). On the other hand, Type V 6 (2.9%) was the least occurring in all quadrants.

**Figure 1:**
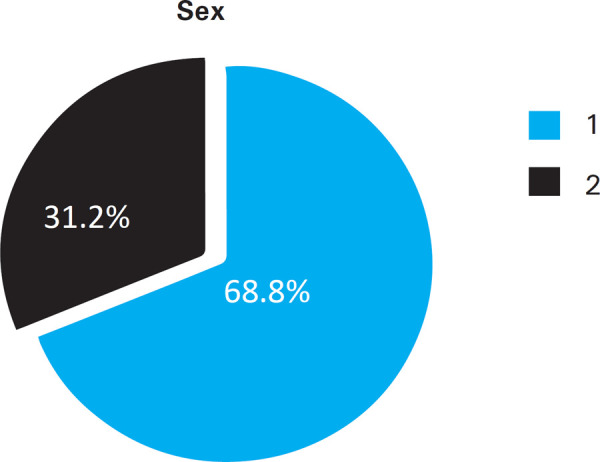
Percentage of Male (1) and Female (2) participants in the study.

Among 205 participants, 141 were males and 64 were females in the age group of 17-24 years (Figure 1).

## DISCUSSION

The quadrant wise findings revealed that, on the right upper quadrant (RUQ), the frequently observed print was Type I (31.7%), followed by Type II (24.4%), Type III (16.1%), Type I’ (15.1%), Type IV (8.3%) and Type V (4.4%) subsequently. While, on left upper quadrant (LUQ), it was Type II (27.8%), followed by Type I (27.3%), Type I’(17%), Type III (12.2%), Type IV (9.8%) and Type V (5.9%). The left lower quadrant (LLQ) followed, the sequential order of Type I (34.1%), Type II (32.2%), Type III (16.6%), Type I’ (9.8%), Type IV (4.4%) and Type V (2.9%). Similarly, Type I (34.1%) was, still the most recurring type, followed by Type II (29.3%), Type III (17.6%), Type I’ (10.2%), Type IV (5.9%) and Type V (2.9%) consequently (Table 1).

**Table 1:** Cumulative distribution of the Types of lip prints among the quadrants.

Lip print Types	RUQ n (%)	LUQ n (%)	LLQ n (%)	RLQ n (%)
I	66 (31.7%)	56 (27.3%)	70 (34.1%)	70 (34.1%)
I′	31 (15.1%)	35 (17%)	20 (9.8%)	21 (10.2%)
II	50 (24.4%)	57 (27.8%)	66 (32.2%)	60 (29.3%)
III	33 (16.1%)	25 (12.2%)	34 (16.6%)	36 (17.6%)
IV	17 (8.3%)	20 (9.8%)	9 (4.4%)	12 (5.9%)
V	9 (4.4%)	12 (5.9%)	6 (2.9%)	6 (2.9%)
Total	205 (100%)	205 (100%)	205 (100%)	205 (100%)

In a study conducted among Goan dental students, Prabhu et al showed in contrast on RUQ, that Type V (52.39%) was the most common followed by Type I′ (17.70%), Type I (14.99%), Type II (10.47%), Type IV (3.61%), Type III (0.81%). Similarly, Type II (27.8%) was the most common, followed by Type I (27.3%), Type I’(17.1%), Type III (12.2%), Type IV (9.8%) and Type V (5.9%) on the LUQ. Whereas, in LLQ, Type V (50.47%) was the most common followed by Type I′ (17.90%), Type I (17.48%), Type II (11.13%), Type IV (2.91%), Type III (0.08%). Moreover, even on the RLQ the pattern in decreasing order was Type V (52.09%), Type I′ (18.72%), Type I (16.68%), Type II (10.97%), Type IV (0.96%), Type III (0.53%).^8^

Furthermore, it can be clearly seen that Type I (34.7%) was frequently encountered in almost all of the quadrants, except the left upper quadrant which was dominated by Type II (27.8%), whereas the least occurring was Type V (2.9%) in all quadrants. Similar results were also seen in studies by Karki^9^, Mohanty et al^10^. Badiye and Kapoor also had similar finding as the predominant Type I (30.63%) whereas Type I’ (1.88%) was the least occurring.^11^ On the contrary, other studies revealed Type IV^12^, Type III^7^ to be frequently common. Another study by Saraswathi revealed Type III and Type IV as the most and least common respectively.^13^ In contrast to our study, Type V was the leading pattern in a study by Prabhu who further sub-classified the Type V.^8^ The variation in the frequency of lip print could be attributed to the race or ethnicity as studies reveal that the predominant pattern seem to vary among population of different origin for e.g. in ours and another study among Nepalese students,^9^ Type I and II was the common and Type V being the least. Similarly Type II was reported to be the most and Types I’ and V to be the least common among Portuguese population^14^ while it was Type III in Egyptian and Malaysian populations^15^ while among Nigerians, Type V was the predominant and Type I’ was the least common.^16^

The lip prints were further considered against quadrant and sexes as shown in Table 2. Amongst male, on right upper quadrant (RUQ), Type I (32.6%) was the most common, followed by Type II (24.8%), Type III (19.9%), Type I’ (10.6%), Type IV (7.8%) and Type V (4.3%). Likewise, studies conducted in Nepal also revealed Type I and II as the most and least common in male and female respectively.^17^ But, in our study, even in female, the Type I (29.7%) was still the most common, followed by Type I’ (25%), Type II (23.4%), Type IV (9.4%), Type III (7.8%) and Type V (4.7%). On the contrary, another study found Type III (34%, 38%) and Type IV (6%, 8%) to be the most and least common in both male and female respectively. ^13^

**Table 2:** Distribution of Lip print Types with respect to sex and quadrant.

Sex	Quadrant			Lip Print Type n (%)			
		I	I′	II	III	IV	V
Male	RUQ	46 (32.6%)	15 (10.6%)	35 (24.8%)	28 (19.9%)	11 (7.8%)	6 (4.3%)
Female		19 (29.7%)	16 (25%)	15 (23.4%)	5 (7.8%)	6 9.4%)	3 (4.7%)
Male	LUQ	39 (27.7%)	20 (14.2%)	38 (27.0%)	21 (14.9%)	13 (9.2%)	10 (7.1%)
Female		17 (26.6%)	15 (23.4%)	19 (29.7%)	4 (6.3%)	7 (10.9%)	2 (3.1%)
Male	LLQ	39 (27.7%)	10 (7.1%)	49 (34.8%)	31 (22.0%)	7 (5%)	5 (3.5%)
Female		31 (48.4%)	10 (15.6%)	17 (26.6%)	3 (4.7%)	2 (3.1%)	1 (1.6%)
Male	RLQ	43 (30.5%)	10 (7.1%)	44 (31.2%)	30 (21.3%)	9 (6.4%)	5 (3.5%)
Female		27 (42.2%)	11 (17.2%)	16 (25.0%)	6 (9.4%)	3 (4.7%)	1 (1.6%)

In left upper quadrant (LUQ), the descending order of lip print in male was Type I (27.7%), Type II (27%), Type III (14.9%), Type I’ (14.2%), Type IV (9.2%) and Type V (7.1%). While in female, it was Type II (29.7%), Type I (26.6%), Type I’ (23.4%), Type IV (10.9%), Type III (6.3%) & V (3.1%). In another study from Nepal, Type I was found to be the most common in both sexes in.^17^ On the contrary, Type I (10%) and Type IV (10%) were the least common whereas Type III (32%, 48%) was the most common in both male and female respectively. ^13^

In left lower quadrant (LLQ), the order in male was, Type II (34.8%), Type I (27.7%), Type III (22%), Type I’ (7.1%), Type IV (5%) and Type V (3.5%) consecutively. In female, it was Type I (48.4%), Type II (26.6%), Type I’ (15.6%), Type III (4.7%), Type IV (3.1%) and Type V (1.6%). However, Type I was still the most common in both sexes in this quadrant too.^17^ in comparison to a study where to Type II (4%, 10%) was the least and Type III (50%, 38%), the most common in both male and female respectively.^13^

In right lower quadrant (RLQ), Type II (31.2%) was the predominating, following which were Type I (30.5%), Type III (21.3%), Type I’ (7.1%), Type IV (6.4%) and Type V (3.5%) in male and Type I (42.2%), Type II (25%), Type I’ (17.2%), Type III (9.4%), Type IV (4.7%) and Type V (1.6%) in female. Another study among Nepalese medical students too revealed comparable findings which stated, Type V was rare while Type I (38%) was leading among male and Type II (42.5%) among female.^9^ Mishra et al showed, Type III (42%), Type II (24%) and Type IV (12%, 20%) to be the most and least common in both male and female respectively.^13^

A study from Libya, on the other hand revealed the commonest lip print pattern to be Type I in 53.37% males and 60.07% females, while Type I to be the least.^18^ Limited sample size along with manual examination of the record can limit the study. Digital examination is likely to produce reliable results.

## CONCLUSIONS

Overall, Type I and Type II were the commonly observed pattern while Type V was the least common. The predominant pattern among male was found to be Type I in RUQ, LUQ and Type II in LLQ, RLQ. Like wise among female, Type I predominated in all the quadrants except LUQ. The least occurring pattern was Type V in all quadrants in both sexes. Despite following the almost similar pattern in predominance and minority of the lip prints in both sexes, there still was variation in frequency and distribution according to the quadrant and sex.

## References

[1] Venkatesh R, David MP (2011). Cheiloscopy: An aid for personal identification. J Forensic Dent Sci.

[2] Prabhu R V, Dinkar AD, Prabhu VD, Rao PK (2012). Cheiloscopy: revisited. J Forensic Dent Sci.

[3] Kaul R, Padmashree SM, Shilpa PS, Sultana N, Bhat S (2015). Cheiloscopic patterns in Indian population and their efficacy in sex determination: A randomized cross-sectional study. J Forensic Dent Sci.

[4] Tandon A, Srivastava A, Jaiswal R, Patidar M, Khare A (2017). Estimation of gender using cheiloscopy and dermatoglyphics. Natl J Maxillofac Surg.

[5] Sharma NA, Eldomiaty MA, Gutiérrez-Redomero E, George AO, Garud RS, Sánchez-Andrés A (2014). Diversity of human lip prints: a collaborative study of ethnically distinct world populations. Ann Hum Biol.

[6] Vats Y, Dhall JK, Kapoor A (2012). Gender variation in morphological patterns of lip prints among some north Indian populations. J Forensic Dent Sci.

[7] Suzuki K, Tsuchihashi Y (1974). A new Attempt of Personal Identification by Means of Lip Print. J Can Soc Forensic Sci. 1971;4:4, 154-158, Studies on personal identification by means of lip prints. Forensic Sci Int.

[8] Prabhu RV, Dinkar A, Prabhu V (2012). A study of lip print pattern in Goan dental students – A digital approach. J Forensic Leg Med.

[9] Karki RK (2012). Lip prints: an identification aid. Kathmandu Univ Med J.

[10] Mohanty B, Jadon AK, Rathore S, Bhatt P, Gupta S, Vaid S (2015). Cheiloscopy As a Means of Personal Identification in Forensic Dentistry: A Study. I J Prev Clin Dent Res.

[11] Kapoor N, Badiye A (2017). A study of distribution, sex differences and stability of lip print patterns in an Indian population. Saudi J Biol Sci.

[12] Verghese AJ, Somasekar M, Babu UR (2001). A Study on Lip Print Types among the People of Kerala. J Indian Acad Forensic Med.

[13] Mishra G, Ranganathan K, Saraswathi T (2009). Study of lip prints. J Forensic Dent Sci.

[14] Costa VA, Caldas IM (2012). Morphologic Patterns of Lip Prints in a Portuguese Population: A Preliminary Analysis. J Forensic Sci.

[15] Abdel Aziz MH, Badr El Dine FMM, Saeed NMM (2016). Regression equations for sex and population detection using the lip print pattern among Egyptian and Malaysian adult. J Forensic Leg Med.

[16] Taura M, Adamu L, Dahiru A, Hamman W, Ibrahim A, Ojo S (2012). Association of lip print and sex among Nigerians. Niger J Basic Clin Sci.

[17] Ghimire N, Nepal P, Upadhyay S, Budhathoki S, Subba A (2014). Lip print pattern: an identification tool. Heal Renaiss.

[18] Peeran S, Naveen Kumar P, Abdalla K, Azaruk FA, Manipady S, Alsaid F (2015). A study of lip print patterns among adults of Sebha city, Libya. J Forensic Dent Sci.

